# Supplementary material for: Psychosocial factors and hospitalisations for COVID-19: Prospective cohort study of the general population
Source: medRxiv. 2020 Jun 1:2020.05.29.20100735. Preprint. [Version 1] doi: 10.1101/2020.05.29.20100735 (PMC7302298; doi:10.1101/2020.05.29.20100735)
Supplement: 1 [file NIHPP2020.05.29.20100735-supplement-1.pdf]

medRxiv preprint doi: https://doi.org/10.1101/2020.05.29.20100735.this version posted June 1, 2020. The copyright holder for this preprint
(which was not certified by peer review) is the author/funder, who has granted medRxiv a license to display the preprint in perpetuity.
It is made available under a CC-BY 4.0 International license .

Supplemental Table 1. Analyses of apparent known risk factors (based on clinical studies) for COVID-19 hospitalisation in UK Biobank (N=431,052)

	All COVID-19 cases (N=908)	COVID-19 cases known to have been hospitalised ('origin'=1) (N=751)	COVID-19 cases known to have been hospitalised ('origin'=1) *and* with >=2 tests conducted (N=445)
Age			
40-49	Ref (1.0)	Ref (1.0)	Ref (1.0)
50-59	0.69 (0.57, 0.83)	0.77 (0.63, 0.95)	0.81 (0.62, 1.05)
60+	1.09 (0.93, 1.27)	1.16 (0.97, 1.38)	1.17 (0.83, 1.48)
Per decade increase	1.15 (1.06, 1.25)	1.18 (1.08, 1.29)	1.17 (1.04, 1.32)
Sex			
Female	Ref (1.0)	Ref (1.0)	Ref (1.0)
Male	1.53 (1.35, 1.75)	1.56 (1.35, 1.81)	1.55 (1.28, 1.87)
Ethnicity			
White	Ref (1.0)	Ref (1.0)	Ref (1.0)
Non-white	2.65 (2.20, 3.20)	2.45 (1.98, 3.03)	3.12 (2.42, 4.01)
Long-standing illness			
No	Ref (1.0)		
Yes	2.06 (1.80, 2.35)	2.03 (1.75, 2.04)	2.26 (1.87, 2.73)

Supplemental Table 2. Odds ratios (95% CI) for the relation of socioeconomic factors with COVID-19 hospitalisation – *based on complete data*

	Case no./ Risk no.[1]	Adjustments					
		**Age & sex**	**Age, sex & ethnicity**	**Age, sex, ethnicity & comorbidity[1]**	**Age, sex, ethnicity & lifestyle factors[2]**	**Age, sex, ethnicity & psychological factors[3]**	**Adjusted for all covariates**
**Educational attainment**							
University Degree	90/55905	1.0 (ref)	1.0 (ref)	1.0 (ref)	1.0 (ref)	1.0 (ref)	1.0 (ref)
Other qualifications	144/77597	1.17 (0.89, 1.52)	1.19 (0.91, 1.55)	1.15 (0.88, 1.49)	1.03 (0.79, 1.34)	1.06 (0.81, 1.39)	0.94 (0.71, 1.24)
No qualifications	54/19237	1.70 (1.20, 2.41)	1.75 (1.23, 2.48)	1.60 (1.12, 2.27)	1.28 (0.89, 1.84)	1.30 (0.89, 1.90)	1.01 (0.68, 1.49)
P for trend		0.005	0.003	0.015	0.245	0.214	0.945
**Annual household income**							
<£18,000	68/26578	1.90 (1.31 2.77)	1.79 (1.23, 2.60)	1.63 (1.12, 2.38)	1.41 (0.96, 2.08)	1.41 (0.95, 2.09)	1.15 (0.77, 1.73)
£18,000-£30,999	71/34301	1.52 (1.06, 2.20)	1.47 (1.02, 2.11)	1.41 (0.98, 2.03)	1.28 (0.89, 1.85)	1.28 (0.88, 1.86)	1.15 (0.79, 1.68)
£31,000-£51,999	60/36526	1.19 (0.82, 1.72)	1.16 (0.80, 1.68)	1.14 (0.79, 1.65)	1.07 (0.74, 1.56)	1.09 (0.75, 1.57)	1.02 (0.70, 1.48)
≥£52,000	54/38368	1.0 (ref)	1.0 (ref)	1.0 (ref)	1.0 (ref)	1.0 (ref)	1.0 (ref)
P for trend		<0.0001	0.001	0.006	0.053	0.060	0.401
**Townsend Deprivation Index**							
1 (low)	69/48529	1.0 (ref)	1.0 (ref)	1.0 (ref)	1.0 (ref)	1.0 (ref)	1.0 (ref)
2	102/53718	1.34 (0.99, 1.82)	1.31 (0.97, 1.78)	1.29 (0.95, 1.75)	1.24 (0.91, 1.68)	1.28 (0.94, 1.74)	1.22 (0.89, 1.65)
3	121/51407	1.70 (1.26, 2.29)	1.55 (1.14, 2.09)	1.46 (1.08, 1.97)	1.28 (0.94, 1.75)	1.41 (1.04, 1.92)	1.20 (0.87, 1.63)
P for trend		<0.0001	0.005	0.016	0.130	0.030	0.297
**Occupational classification**							
Managers, senior officials, etc	154/76773	1.0 (ref)	1.0 (ref)	1.0 (ref)	1.0 (ref)	1.0 (ref)	1.0 (ref)
Administrative, secretarial, etc	42/29676	0.71 (0.51, 1.01)	0.71 (0.51, 1.01)	0.70 (0.50, 0.99)	0.65 (0.46, 0.91)	0.64 (0.45, 0.90)	0.59 (0.42, 0.84)
Personal service, sales, etc	48/21630	1.12 (0.81, 1.55)	1.08 (0.78, 1.50)	1.04 (0.75, 1.45)	0.90 (0.65, 1.25)	0.86 (0.61, 1.21)	0.75 (0.53, 1.06)
P for trend		0.993	0.887	0.713	0.219	0.145	0.027

[1]Comorbidity includes diagnoses of vascular or heart disease, diabetes, chronic bronchitis or emphysema, asthma, and hypertension defined according to measured blood pressure and/or use of anti-hypertensive medication. [2] Lifestyle factors includes body mass index, smoking status, alcohol intake frequency & number of types of physical activity taken in last four weeks. [2] Psychological factors include psychological distress, psychiatric consultation, neuroticism, verbal and numerical reasoning, & reaction time.

medRxiv preprint doi: https://doi.org/10.1101/2020.05.29.20100735.this version posted June 1, 2020. The copyright holder for this preprint (which was not certified by peer review) is the author/funder, who has granted medRxiv a license to display the preprint in perpetuity.
It is made available under a CC-BY 4.0 International license .

Supplemental Table 3. Odds ratios (95% CI) for the relation of psychological factors with COVID-19 hospitalisation – *based on complete data*

	Case no./ Risk no.[1]	Adjustments					
		Age & sex	Age, sex & ethnicity	Age, sex, ethnicity & comorbidity[1]	Age, sex, ethnicity & lifestyle factors[2]	Age, sex, ethnicity & socioeconomic factors[3]	Adjusted for all covariates
**Psychological distress**							
1 (low)	149/96723	1.0 (ref)	1.0 (ref)	1.0 (ref)	1.0 (ref.)	1.0 (ref.)	1.0 (ref)
2	161/93337	1.14 (0.91, 1.42)	1.15 (0.92, 1.44)	1.12 (0.89, 1.40)	1.09 (0.87, 1.36)	1.13 (0.91, 1.42)	1.07 (0.86, 1.34)
3	113/555059	1.36 (1.06, 1.75)	1.32 (1.03, 1.70)	1.25 (0.97, 1.61)	1.15 (0.89, 1.48)	1.22 (0.95, 1.57)	1.09 (0.84, 1.40)
P for trend		0.016	0.028	0.080	0.272	0.107	0.501
Per SD increase		1.18 (1.08, 1.29)	1.16 (1.06, 1.27)	1.13 (1.03, 1.24)	1.09 (1.00, 1.20)	1.12 (1.02, 1.22)	1.07 (0.97, 1.17)
**Psychiatric consultation**							
No	412/238768	1.0 (ref)	1.0 (ref)	1.0 (ref)	1.0 (ref)	1.0 (ref)	1.0 (ref)
Yes	58/26798	1.26 (0.96, 1.67)	1.29 (0.98, 1.70)	1.26 (0.95, 1.66)	1.19 (0.90, 1.57)	1.22 (0.92, 1.61)	1.15 (0.87, 1.52)
**Neuroticism**							
1 (low)	119/69458	1.0 (ref)	1.0 (ref)	1.0 (ref)	1.0 (ref)	1.0 (ref)	1.0 (ref)
2	191/109358	1.03 (0.82, 1.30)	1.05 (0.83, 1.32)	1.03 (0.81, 1.29)	1.02 (0.81, 1.28)	1.03 (0.82, 1.29)	1.01 (0.80, 1.27)
3	157/85968	1.09 (0.85, 1.39)	1.11 (0.87, 1.41)	1.06 (0.83, 1.36)	1.03 (0.81, 1.32)	1.06 (0.83, 1.35)	1.00 (0.79, 1.28)
P for trend		0.491	0.395	0.612	0.804	0.660	0.982
Per SD increase		1.03 (0.94, 1.13)	1.04 (0.94, 1.14)	1.02 0.93, 1.12)	1.00 (0.91, 1.10)	1.01 (0.92, 1.11)	0.99 (0.90 1.09)
**Verbal numerical reasoning**							
1 (low)	81/26374	2.52 (1.83, 3.47)	2.09 (1.50, 2.93)	2.03 (1.45, 2.84)	1.86 (1.33, 2.62)	2.06 (1.44, 2.96)	1.98 (1.38, 2.84)
2	86/41404	1.70 (1.24, 2.34)	1.61 (1.17, 2.21)	1.59 (1.16, 2.18)	1.53 (1.11, 2.11)	1.60 (1.16, 2.21)	1.58 (1.14, 2.18)
3	70/57112	1.0 (ref)	1.0 (ref)	1.0 (ref)	1.0 (ref)	1.0 (ref)	1.0 (ref)
P for trend		<0.0001	<0.0001	<0.0001	<0.0001	<0.001	<0.0001
Per SD decrease		1.46 (1.27, 1.66)	1.34 (1.17, 1.54)	1.32 (1.15, 1.52)	1.27 (1.10, 1.46)	1.33 (1.15, 1.55)	1.31 (1.12, 1.52)
**Reaction time**							
1 (low)	164/99395	1.0 (ref)	1.0 (ref)	1.0 (ref)	1.0 (ref)	1.0 (ref)	1.0 (ref)
2	155/88713	1.09 (0.87 1.36)	1.05 (0.84, 1.31)	1.04 (0.84, 1.31)	1.03 (0.82, 1.29)	1.03 (0.82, 1.29)	1.02 (0.81, 1.28)
3	148/76914	1.21 (0.96, 1.53)	1.10 (0.87, 1.39)	1.09 (0.86, 1.37)	1.05 (0.83, 1.33)	1.04 (0.82, 1.31)	1.02 (0.80, 1.29)
P for trend		0.104	0.424	0.496	0.659	0.759	0.869
Per SD increase		1.16 (1.06, 1.27)	1.10 (1.01, 1.21)	1.09 (1.00, 1.20)	1.08 (0.99, 1.19)	1.07 (0.98, 1.17)	1.06 (0.97, 1.17)

[1] Comorbidity includes diagnoses of vascular or heart disease, diabetes, chronic bronchitis or emphysema, asthma, and hypertension defined according to measured blood pressure and/or use of anti-hypertensive drugs. [2] Lifestyle factors included body mass index, smoking status, alcohol intake frequency & number of types of physical activity taken in last four weeks. [3] Socioeconomic factors included occupational classification, highest educational attainment, Townsend deprivation index, & household income before tax

medRxiv preprint doi: https://doi.org/10.1101/2020.05.29.20100735.this version posted June 1, 2020. The copyright holder for this preprint (which was not certified by peer review) is the author/funder, who has granted medRxiv a license to display the preprint in perpetuity. It is made available under a CC-BY 4.0 International license .

medRxiv preprint doi: https://doi.org/10.1101/2020.05.29.20100735.this version posted June 1, 2020. The copyright holder for this preprint (which was not certified by peer review) is the author/funder, who has granted medRxiv a license to display the preprint in perpetuity.
It is made available under a CC-BY 4.0 International license .

Supplementary Table 4. Odds ratios (95% CI) for the relation of socioeconomic factors with COVID-19 hospitalisation – *impact of adjusting for biomarkers*

		Adjustments		
	**Case no./Risk no.**[1]	**Age, sex & ethnicity**	**Age, sex, ethnicity & biomarker**[2]	**All covariates**[3]
**Educational attainment**		N=420502	N=301981	N=108462
Degree	229/137717	1.0 (ref)	1.0 (ref)	1.0 (ref)
Other qualifications	406/214337	1.19 (1.01, 1.41)	1.15 (0.93, 1.41)	0.94 (0.66, 1.33)
No qualifications	241/70003	2.07 (1.71, 2.50)	1.82 (1.43, 2.31)	1.10 (0.68, 1.77)
P for trend		<0.0001	<0.0001	0.802
**Annual household income**		N=363175	N=261825	N=96562
<£18,000	241/81207	1.89 (1.51, 2.35)	1.47 (1.12, 1.93)	1.03 (0.62, 1.72)
£18,000-£30,999	179/92461	1.27 (1.01, 1.60)	1.09 (0.83, 1.44)	1.04 (0.65, 1.68)
£31,000-£51,999	167/95454	1.17 (0.94, 1.47)	1.10 (0.84, 1.49)	1.13 (0.72, 1.78)
≥£52,000	141/95097	1.0 (ref)	1.0 (ref)	1.0 (ref)
P for trend		<0.0001	0.007	0.988
**Neighbourhood deprivation**		N=427986	N=301418	N=108898
1 (low)	205/143483	1.0 (ref)	1.0 (ref)	1.0 (ref)
2	267/143548	1.29 (1.07, 1.55)	1.14 (0.91, 1.42)	1.10 (0.76, 1.58)
3	436/143517	1.97 (1.66, 2.34)	1.58 (1.28, 1.96)	0.97 (0.66, 1.42)
P for trend		<0.0001	<0.0001	0.840
**Occupational classification**		N=307262	N=221854	N=91388
Managers, senior officials, etc	324/175637	1.0 (ref)	1.0 (ref)	1.0 (ref)
Administrative, secretarial, etc	94/74137	0.69 (0.55, 0.87)	0.65 (0.49, 0.87)	0.48 (0.30,0.77)
Personal service, sales, etc	149/58915	1.30 (1.07, 1.59)	1.28 (1.01, 1.64)	0.75 (0.48, 1.15)
P for trend		0.091	0.223	0.054

[1] Numbers based on unadjusted model. [2]Biomarkers included FEV1, and blood concentrations of c-reactive protein, HbA1c, and HDL cholesterol. [3]Multivariate model included age, sex, ethnicity, diagnoses of vascular or heart disease, diabetes, chronic bronchitis or emphysema, asthma, hypertension defined according to measured blood pressure and/or use of anti-hypertensive drug, body mass index, smoking status, alcohol intake frequency, number of types of physical activity taken in last four week, psychological distress, psychiatric consultation, reasoning, reaction time, FEV1, and blood concentrations of c-reactive protein, HbA1c and HDL cholesterol.

medRxiv preprint doi: https://doi.org/10.1101/2020.05.29.20100735.this version posted June 1, 2020. The copyright holder for this preprint (which was not certified by peer review) is the author/funder, who has granted medRxiv a license to display the preprint in perpetuity.
It is made available under a CC-BY 4.0 International license .

Supplementary Table 5. Odds ratios (95% CI) for the relation of psychological factors with COVID-19 hospitalisation – *impact of adjusting for biomarkers*

	Case no./Risk no.[1]	Adjustments		
		**Age, sex & ethnicity**	**Age, sex, ethnicity & biomarkers[2]**	**All covariates[3]**
**Psychological distress**		N=383655	N=273998	N=179391
1 (low)	267/153504	1.0 (ref)	1.0 (ref)	1.0 (ref)
2	291/140200	1.29 (1.09, 1.53)	1.16 0.94, 1,43)	1.04 (0.79, 1.36)
3	224/91205	1.51 (1.26, 1.81)	1.28 (1.01, 1,61)	0.98 (0.71, 1.34)
P for trend		<0.0001	0.033	0.931
Per SD increase		1.19 (1.12, 1.26)	1.12 (1.04, 1.22)	1.04 (0.93, 1.17)
**Psychiatric consultation**		N=426823	N=303561	N=194162
No	751/379080	1.0 (ref)	1.0 (ref)	1.0 (ref)
Yes	140/487739	1.51 (1.26, 1.81)	1.44 (1.15, 1.82)	1.27 (0.91, 1.76)
**Neuroticism**		N=424212	N=302071	N=193565
1 (low)	224/106910	1.0 (ref)	1.0 (ref)	1.0 (ref)
2	345/174705	1.03 (0.87, 1.22)	0.98 (0.79, 1.22)	1.03 (0.78, 1.37)
3	319/144092	1.21 (1.02, 1.44)	1.19 (0.96, 1.48)	1.06 (0.78, 1.42)
P for trend		0.023	0.088	0.725
Per SD increase		1.08 (1.01, 1.16)	1.08 (0.99 1.17)	0.99 (0.88, 1.11)
**Verbal numerical reasoning**		N=174581	N=122752	N=89129
1 (low)	152/43988	2.31 (1.77, 3.02)	2.35 (1.68, 3.30)	2.08 (1.33, 3.28)
2	115/58446	1.45 (1.10, 1.90)	1.51 (1.07, 2.13)	1.67 (1.12, 2.49)
3	96/72833	1.0 (ref)	1.0 (ref)	1.0 (ref)
P for trend		<0.0001	<0.0001	0.001
Per SD decrease		1.37 (1.23, 1.53)	1.37 (1.19, 1.58)	1.33 (1.10, 1.61)
**Reaction time**		N=424432	N=302492	N=193832
1 (low)	262/140934	1.0 (ref)	1.0 (ref)	1.0 (ref)
2	274/141575	1.00 (0.84, 1.19)	1.01 (0.81, 1.25)	1.09 (0.83, 1.42)
3	345/143368	1.16 (0.98, 1.37)	1.07 (0.87, 1.33)	1.05 (0.79, 1.40)
P for trend		0.078	0.505	0.724
Per SD increase		1.07(1.01, 1.14)	1.06 (0.98, 1.15)	1.08 (0.96, 1.21)

[1] Numbers based on age & sex adjusted model. [2] Biomarkers included FEV1, and blood concentrations of c-reactive protein, HbA1c, and HDL cholesterol. [3]Multivariate model included age, sex, ethnicity, diagnoses of vascular or heart disease, diabetes, chronic bronchitis or emphysema, asthma, hypertension defined according to measured blood pressure and/or use of anti-hypertensive drug, body mass index, smoking status, alcohol intake frequency, number of types of physical activity taken in last four week, occupational classification, educational attainment, Townsend deprivation index, household income before tax, FEV1, and blood concentrations of c-reactive protein, HbA1c and HDL cholesterol.
